# Bilateral ECT induces bilateral increases in regional cortical thickness

**DOI:** 10.1038/tp.2016.139

**Published:** 2016-08-23

**Authors:** P van Eijndhoven, P Mulders, L Kwekkeboom, I van Oostrom, M van Beek, J Janzing, A Schene, I Tendolkar

**Affiliations:** 1 Department of Psychiatry, Radboud University Medical Center, Nijmegen, The Netherlands; 2 Donders Institute for Brain Cognition and Behavior, Centre for Cognitive Neuroimaging, Nijmegen, The Netherlands; 3 Faculty of Medicine and LVR Clinic for Psychiatry and Psychotherapy, University of Duisburg-Essen, Essen, Germany

## Abstract

Electroconvulsive therapy (ECT) is the most effective treatment for patients suffering from severe or treatment-resistant major depressive disorder (MDD). Unfortunately its underlying neurobiological mechanisms are still unclear. One line of evidence indicates that the seizures produced by ECT induce or stimulate neuroplasticity effects. Although these seizures also affect the cortex, the effect of ECT on cortical thickness is not investigated until now. We acquired structural magnetic resonance imaging data in 19 treatment-resistant MDD patients before and after a bilateral ECT course, and 16 healthy controls at 2 time points, and compared changes in cortical thickness between the groups. Our results reveal that ECT induces significant, bilateral increases in cortical thickness, including the temporal pole, inferior and middle temporal cortex and the insula. The pattern of increased cortical thickness was predominant in regions that are associated with seizure onset in ECT. *Post hoc* analyses showed that the increase in thickness of the insular cortex was larger in responders than in non-responders, which may point to a specific relationship of this region with treatment effects of ECT.

## Introduction

Although electroconvulsive therapy (ECT) is the most effective treatment for patients suffering from severe or treatment-resistant major depressive disorder (MDD),^1^ achieving faster and higher response rates than pharmacotherapy,^2^ the underlying neurobiological mechanisms remain poorly understood. Several hypotheses, based on the effects of ECT on monoamine systems and endocrine function, have been proposed.^3^ In explanations of its superior effectiveness, the neuroplasticity effects of ECT have become dominant.

The therapeutic efficacy of ECT is related to its capacity to generate a generalized epileptic seizure.^4^ Animal models of electroconvulsive stimulation have shown that convulsions cause neuroplasticity effects.^5^ ECT-induced seizures engage both cortical and subcortical networks to varying degrees and result in increased cerebral blood blow in focal cortical areas.^6, 7^ In line with these broad hemodynamic effects of ECT on the brain, recent research indicates that next to effects on volume of hippocampus^8, 9, 10^ or amygdala^11, 12^ ECT induces also neuroplasticity effects in the cortex.^9, 13^ Up till now, there are no studies that have used cortical thickness analysis to investigate the effects of ECT treatment, which is a sensitive method to study longitudinal changes in the cortex.^14^

Cortical thickness represents the thickness of the outer layer of gray matter in the brain which varies greatly between species and also between cortical subregions within subjects.^14^ Differences in cortical thickness have been observed in a variety of neuropsychiatric disorders.^15, 16^ Emerging evidence indicates that cortical thickness is affected in MDD, primarily expressed as regional thinning in the cingulate and orbitofrontal cortex^17, 18, 19, 20, 21^ although areas of thickening have also been found.^21, 22^ A recent, large meta-analysis of cortical thickness in 20 depression cohorts worldwide gathered in the ENIGMA group, confirmed thinning of the orbitofrontal cortex and cingulate cortex, and also identified thinning of the insula and temporal lobes.^23^ In contrast with decreases in hippocampal volume, which was mainly associated with recurrent depression,^24^ cortical thickness changes were robustly detectable in adult patients at their first episode.^23^

Based on the fact that ECT-induced seizures affect the cortex, we hypothesize that ECT leads to regional increases in cortical thickness. A secondary goal is to identify changes in cortical thickness that may be related to treatment response.

## Materials and methods

### Subjects

Twenty three patients (8 male/15 female; age 50.7±8.5 years) with treatment-resistant MDD were recruited at the department of psychiatry of the Radboud University Medical Centre Nijmegen. Sample size was based on previous studies investigating the longitudinal effects of ECT on structural measures such as hippocampal^10^ and gray matter^9, 13^ volume. All patients were diagnosed with MDD using the Structural Interview for DSM disorders (SCID) and were eligible to receive ECT treatment based on treatment resistance for medication, according to the Dutch guidelines for depression and ECT.^25^ Briefly this means that patients had failed to respond to a stepwise treatment including serotonin- or serotonin–noradrenaline-reuptake inhibitors, tricyclic antidepressants and augmentation with lithium or anti-epileptics, and in some cases MAO-inhibitors before receiving ECT. Exclusion criteria were bipolar depression, having received ECT within 1 year prior to the current course, schizophrenia or a history of alcohol or substance abuse. Twenty two sex- and age-matched healthy controls (8 male/14 female; mean age=50.8±8.8 years) were recruited from the local area by advertisement. Exclusion criterion for controls was having any life-time psychiatric disorder. Exclusion criteria for all persons included were present or past relevant somatic or neurologic comorbid disorder, and magnetic resonance imaging (MRI)-related exclusion criteria such as claustrophobia, a pacemaker or pregnancy.

Patients were tapered off from all psychotropic medication, such as antidepressants, antipsychotics, mood stabilizers and benzodiazepines at 1 week before the start of ECT. Only incidental use of benzodiazepines or promethazine was permitted during the course of ECT.

During the course of ECT treatment, severity of depression was measured using the Hamilton Depression Rating Scale (HDRS-17).^26^ All participants provided written informed consent and the study protocol was approved by the review board of the Radboud University Nijmegen Medical Centre.

### ECT-series

ECT was administered bilaterally at the temporal window using a brief pulse, constant current apparatus with a maximum stimulus output of 1008 mC (200%) (Thymatron System IV, Somatics, Lake Bluff, IL, USA). Seizure threshold was determined during the first session with stimulus titration. The seizure threshold is defined as the minimum stimulus dosage required to generate a generalized seizure of at least 20-s duration according to the cuff method. For the second treatment session, a stimulus intensity of 1.5 times the initial seizure threshold was used. Global anesthesia was achieved by administering i.v. etomidate (0.2–0.3 mg kg^−1^), followed by succinylcholine 1.0 mg kg^−1^ to achieve muscle relaxation. Patients received treatment twice a week for as long as there was substantial improvement in symptom severity. When no improvement was measured after at least 10 adequate treatment sessions or no further improvement during the last 4 sessions, treatment was discontinued. On average, patients were treated 18±7 times (range 7–32 sessions).

### Procedure

Patients were assessed at two time points: T1, 1 week before the first ECT treatment; and T2, within 1 week of completing the ECT series. Of the 23 patients examined at T1, 19 patients completed T2. Drop out was caused by defect scanner (two patients) and to patient-initiated discontinuation of ECT (two patients). Of the 22 healthy controls that were examined at T1, 16 were re-examined at T2, with a time interval similar to the mean of the ECT series, to control for timing and test–retest effects. The longitudinal effects of ECT were analyzed by comparing changes in cortical thickness between the patients and controls with data for both T1 and T2.

### Imaging technique and cortical thickness measurements

High-resolution MPRAGE images were acquired (1.5 T Avanto, Siemens, Erlangen, Germany) for all subjects. Acquisition parameters were: T1 850 (ms), TR 2250 (ms), TE 3.68 (ms), flip angle 15 (°), FoV 256 × 256 × 176 (mm), voxel-size 1.0 × 1.0 × 1.0 (mm).

The MRI data were analyzed by using FreeSurfer software version 5.3.0 (http://surfer.nmr.mgh.harvard.edu). This software package is almost completely automated and reliably computes cortical thickness. The data were motion corrected and intensity normalized. We performed segmentation of white matter and tessellation of the gray–white matter junction. Topological defects in the gray–white estimate were fixed. Then a deformable surface algorithm was applied to find the pial surface. We visually inspected the entire cortex in each subject and corrected any inaccuracies in segmentation manually. The reconstructed cortical surfaces were inflated to normalize interindividual differences in gyral or sulcal depth. Each reconstructed brain was morphed and registered to an average spherical surface representation so that sulci and gyri were optimally aligned and cortical thickness difference maps could be constructed on a common spherical coordinate system.

To extract reliable thickness estimates, images of subjects were automatically processed within the longitudinal stream in FreeSurfer.^27^ Specifically an unbiased within-subject template space and image was created using robust, inverse consistent registration.^28^ Several processing steps, such as skull stripping, Talairach transforms, atlas registration, as well as spherical surface maps and parcellations were then initialized with common information from the within-subject template, significantly increasing reliability and statistical power.^27^

### Statistical analysis

After smoothing (full width at half maximum, 10 mm^21^), the cortical thickness data were averaged across subjects in the spherical coordinate system, so that surface areas with significant differences of mean cortical thickness could be overlaid in statistical difference maps (using *t*-statistics). We addressed cross-sectional differences between the MDD patients and the healthy controls at baseline, corrected for age and sex. Longitudinal changes between the two time points for the two groups were analyzed with the general linear model functionality within QDEC, FreeSurfer's graphical interface for analyzing group data. We estimated for the whole brain (in vertex-wise statistical difference maps) the main effect of group (ECT versus healthy controls), on symmetrized percent change of the cortical thickness corrected for age and sex. Correction for multiple comparisons was applied by clusterwise correction, based on Monte Carlo *Z* simulation, build into QDEC (threshold 0.005, sign absolute).^29^

In a second step, we compared responders (*n*=10) and non-responders (*n*=9) to ECT by a whole-brain analysis. In addition to this exploratory whole-brain analysis looking into effects of successful treatment, we also performed a *post hoc* analysis with independently defined cortical parcellations (from the Desikan–Killiany Atlas), to explore whether there were effects associated with response (defined as a decrease in HDRS of >50%), within the regions that showed the longitudinal increases in the first step (that is, insula, temporal pole and temporal cortex). Changes in mean thickness of these parcellations were analyzed with SPSS (Statistic Package for the Social Sciences) software version 20.0 (Armonk, NY, USA), with independent *t*-tests. Further, these absolute changes in thickness were used for exploratory correlational analyses (Pearson's correlations, *P*<0.05 one-tailed) with clinical variables: age, change in Hamilton depression score and parameters linked to the ECT course: that is, number of ECT sessions, change in charge between last and first session and change in seizure duration between first and last session.

## Results

Patient characteristics are summarized in Table 1. There were no significant differences between MDD patients and healthy controls in age, sex distribution, level of education or handedness. Hamilton scores after (12.6±7.1) on average 16.9±6.2 ECT sessions differed significantly from Hamilton scores (21.9±5.3) before ECT (*P*<0.001). HDRS based response rate was 57%. There were no differences in number of ECT sessions between responders and non-responders (*P*=0.93).

Additional information about the clinical characteristics of each patient, such as age at onset, number of depressive episodes, duration of the current episode, melancholic and psychotic features and details about medication history is presented in Supplementary Table 1A. Details about the ECT course of each patient are provided in Supplementary Table 1B (number of ECT sessions, pulse width, charge at start and end of ECT course, seizure duration at start and end). There was a significant difference between both charge and seizure duration at the start and end of the ECT course (mean charge start 175 mC; end 393 mC; *P*<0.001; mean seizure duration start=52 s; end 36; *P*<0.001).

### Cross-sectional effects

Whole-brain comparisons for baseline cortical thickness between MDD patients and healthy controls revealed no significant differences between the two groups.

### Longitudinal effects of ECT

**Figure 1:**
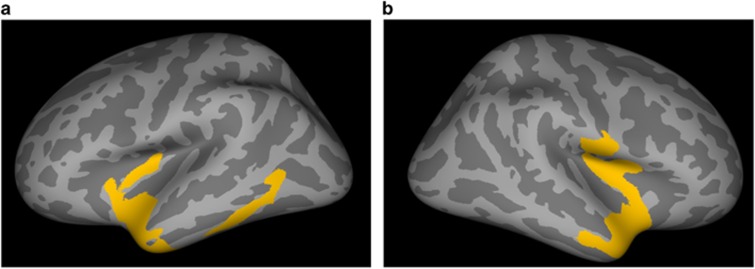
Increases in cortical thickness in MDD patients (*n*=19) during ECT treatment in comparison with healthy controls (*n*=16) on inflated brain. Shown is the statistical output of QDEC, the graphical interface of Freesurfer, with the results corrected for multiple comparisons by Monte Carlo *Z* simulation*. (**a**) Left hemisphere, with a large cluster in yellow extending from temporal pole, middle and superior temporal cortex to the insula and the inferior temporal cortex. (**b**) Right hemisphere, with a large cluster in yellow extending from the temporal pole to the insula. *Monte Carlo Null-*Z* simulation, threshold 0.005 (absolute). MDD, major depressive disorder.

Whole-brain comparisons revealed large bilateral clusters (left 2886 mm^2^, right 3599 mm^2^) of increased thickness after ECT treatment. These clusters extended from the temporal pole, middle and superior temporal cortex to the insula and inferior temporal cortex in the left hemisphere (see Figure 1 and Table 2). There were no areas showing a significant decrease of cortical thickness in the longitudinal analysis, neither were there changes in cortical thickness in the healthy controls in the longitudinal analysis.

### Relations between increase in cortical thickness, treatment response and clinical variables

The whole-brain analysis did not give rise to significant differences in cortical thickness between responders and non-responders as a function of ECT treatment. In a next step, we took the mean thickness of predefined cortical parcellations, which were included within the bilateral cluster of increased cortical thickness, and compared responders and non-responders to ECT. Supplementary Table 2 presents the absolute increases of the predefined cortical parcellations that were identified in the second step. Independent *t*-tests of changes in thickness of both the left and the right insula revealed significant differences between responders and non-responders; responders showed a larger increase in cortical thickness than non-responders (left insula; *P*=0.017, and right insula; *P*=0.017). Changes in cortical thickness of the temporal pole and temporal cortex (inferior, middle and superior) did not differ between responders and non-responders.

Exploratory correlational analyses with clinical variables revealed that the change in HDRS score was negatively correlated with change in mean cortical thickness of the right insula (*r*=−0.45; *P*=0.028; one-tailed). The number of ECT sessions was positively correlated with change in mean thickness of the left temporal pole (*r*=0.43; *P*=0.035 one-tailed), the left middle temporal cortex (*r*=0.51; *P*=0.013 one-tailed) and the left inferior temporal cortex (*r*=0.45; *P*=0.028 one-tailed). There were no significant correlations with age and change in seizure duration or change in electrical charge between the first and last ECT session.

## Discussion

In this study, we believe we show for the first time that ECT induces significant increases in cortical thickness in treatment-resistant MDD patients. We used bilateral ECT, which induced large bilateral increases in cortical thickness, including the temporal pole, the inferior and middle temporal cortex and the insula. *Post hoc* analyses revealed that increased thickness of the bilateral insular cortex differentiated responders and non-responders to ECT, which may point to a specific relationship of this region with treatment effects. Below we will discuss these findings in more detail.

The widespread increases in cortical thickness that were revealed in this study extend the existing animal and human data on neuroplastic effects of ECT, pointing to broader neuroplastic effects of ECT in MDD, beyond effects in the hippocampus and amygdala that were reported before.^8, 11, 12, 30^ Although neuroimaging data do not allow us to directly investigate the exact nature of these neuroplastic effects, it can be assumed that increases in cortical thickness could reflect changes in neurons, glia cells or neuropil.^31^ Increased cortical thickness was predominant in regions such as temporal cortex that are subjected to the highest electric field strength and may therefore be associated with seizure onset.^6, 7^ Previous research has found direct effects of ECT on cerebral blood flow^32^ and gray matter volume voxel based morphometry^9, 13^ in these regions and this pattern suggests that the changes are a direct consequence of seizure onset that is induced by ECT.

In the course of treatment, most patients show an increase in seizure threshold and a decrease in seizure duration,^33, 34^ pointing to additional anticonvulsive properties of ECT that may also count for its clinical effectiveness.^33, 35^ Speculatively, the increased cortical thickness could drive these anticonvulsant properties of ECT, possibly by means of an increase in GABAergic inhibitory interneurons or glia cells, in line with the anticonvulsant mechanisms of ECT.^33, 35^ Previous reports have found increases in cortical and serum GABA levels in patients following ECT treatment,^36, 37^ which in turn increase the expression of neurotrophic factors such as brain-derived neurotrophic factor.^38^ Animal models of ECT have indicated that the neuroplasticity effects of ECT are mediated by increases in vascular endothelial growth factor and brain-derived neurotrophic factor.^39, 40, 41^ Increased GABAergic neurotransmission could also be relevant therapeutically via restoration of cortical control over hyperactive limbic structures, through a process known as cortical inhibition.^42^ ECT patients in our sample showed an increase in charge between the first and last ECT session and a decrease in seizure duration, which may point to anticonvulsive properties of ECT. Additional correlational analysis with these parameters and the changes in cortical thickness could not establish a direct relation of increases cortical thickness with anticonvulsant properties of ECT.

When comparing responders and non-responders to ECT, we found a response-related effect in the bilateral insular cortex. Though still explorative in nature, this increase could be a response marker for therapeutic effects of ECT. These findings add to the increasing amount of evidence implicating the insular region as an important structure in the pathophysiology of MDD.^43, 44, 45, 46^ The insula monitors internal states,^47^ is involved in emotional and sensorimotor processing and has extensive connections with default mode network regions.^48^ MDD is associated with decreased interoceptive activity and altered activity and connectivity in the insula^49^ and earlier research has found strong gray matter reductions in the insular cortex in MDD patients.^45^ Several studies have identified the insula as an neural correlate of treatment response in MDD.^48^ An inability to control internal emotional states could explain core symptoms of depression and restoration of this function would potentially explain part of the treatment effect of ECT.

Our study is limited by the relatively small sample size, which may account for the absence of baseline differences between MDD patients and matched controls, and which makes it also difficult to establish differences between responders and non-responders. While this should be a clear goal of future studies, we would like to emphasize that patients were otherwise homogenous in terms of diagnosis and free of any medication that may have interfered with potential effects of ECT. We found bilateral changes in cortical thickness associated with the method of bilateral stimulation, which is in line with increases in gray matter volume in the temporal cortex that were revealed by Ota *et al.*^13^ In contrast, right unilateral stimulation seems to induce a different pattern of neuroplastic changes. Abbott *et al.*^10^ found that right unilateral ECT induced a right-sided increase in hippocampal volume and connectivity after ECT and Dukart *et al.*^9^ reported an increase in gray matter volume in the right anterior temporal pole and insula. At odds with these lateralization effects is the study by Joshi *et al.*^12^ who found bilateral increases in both hippocampal and amygdala volumes in a sample of patients who received predominantly right unilateral ECT. Possibly, not the stimulation method *per se*, but rather the capacity of electrical stimulation to induce a generalized seizure may determine the pattern of neuroplastic responses. Also it not clear what determines the differences between the hemispheres that are apparent in Figures 1a and b. Another limitation is that we do not know yet whether these increases in cortical thickness reflect a temporary effect, as was shown for the increase in volume of the hippocampus induced by ECT^50^ and longer follow-up studies in new line of ECT neuroimaging studies are certainly warranted.

In summary, we show that ECT does not only change the neuroplasticity of subcortical brain regions, but also leads to regional increases in cortical thickness. The localization of this increase suggests that it is related to seizure onset in ECT. Moreover, extension of this area of increase to the insula seems to be an important factor, which may determine the therapeutic response. The exact nature of these changes has to be investigated by future research in larger samples and multimodal imaging techniques including magnetic resonance spectroscopy. Further it should be established whether the ECT-induced changes in cortical thickness remain on follow-up.

## Figures and Tables

**Table 1:** Demographical and clinical characteristics of the MDD patients and matched healthy control

	*Patients (*n=*23)*	*Healthy (*n=*22)*	*Group difference* P^a^
Age (years)	50.7 (8.5)	50.8 (8.8)	0.98
Sex (male/female)	8/15	8/14	0.91^b^
Education (1–5)^c^	3.2 (0.9)	3.4 (0.8)	0.58
Handedness (right/left)	21/2	19/3	0.60^b^
HDRS 17 baseline	21.9 (5.3)	—	—
HDRS 17 after ECT	12.6 (7.1)	—	—
Responders (% of patients)	13 (57%)	—	—
Age at onset (years)	40.8 (10.9)	—	—
Number of depressive episodes	2.8 (1.5)	—	—
Duration current episode (months)	31.0 (52.7)	—	—
Psychotic features (present/not present)	6/17	—	—
Melancholic features (present/not present)	15/8	—	—
ECT sessions	16.9 (6.2)	—	—

Abbreviations: ECT, electroconvulsive therapy; HDRS, Hamilton Depression Rating Scale 17 item; MDD, major depressive disorder.

Data are expressed as mean (s.d.) unless otherwise specified.

a Independent *t*-test.

b Pearson's *X*^2^ for categorical variables.

c Educational level is coded level 1–5 (5=academic), according to the Dutch education system.

**Table 2:** Longitudinal changes in cortical thickness (SPC) in MDD patients during ECT treatment (*n*=19) in comparison with healthy controls (*n*=16)

	*Size (mm*^*2*^)	*Talairach (*x y z*)*	*Number of vertices*	*Clusterwise* P^a^
*MDD>healthy controls, left hemisphere*				
Temporal pole	2886	−28 6 –34	5527	0.0001
Middle temporal cortex	791	−58 −56 −2	1178	0.0001
				
*MDD>healthy controls, right hemisphere*				
Insula	3599	37 −13 2	7970	0.0001

Abbreviations: ECT, electroconvulsive therapy; MDD, major depressive disorder; SPC, symmetrized percent change.

a Monte Carlo null-*Z* simulation, threshold 0.005 (absolute).

## Supplementary Material

Supplementary Table 1A

Supplementary Table 1B
