# Supplementary material for: Bilateral ECT induces bilateral increases in regional cortical thickness
Source: Transl Psychiatry. 2016 Aug 23;6(8):e874–. doi: 10.1038/tp.2016.139 (PMC5022085; doi:10.1038/tp.2016.139)
Supplement: Supplementary Table 1A [file tp2016139x1.docx]

| Patient | Gender | Age | Age at onset | Number of depressive episodes | Duration current episode (months) | Psychotic features | Melancholic features | HDRS | SSRI | SNRI | TCA | Other AD | Lithium augmentation | MAO-Inhibitor | Anti-epileptic | Atypical antipsychotic | Previous ECT |
| --- | --- | --- | --- | --- | --- | --- | --- | --- | --- | --- | --- | --- | --- | --- | --- | --- | --- |
| 1 | m | 55 | 33 | 1 | 264 | no | no | 14 | 2 | 1 | 3 | 4 | 1 | 3 | 2 | 1 | no |
| 2 | f | 54 | 52 | 6 | 18 | no | yes | 24 | 2 | 1 | 3 | 0 | 1 | 0 | 1 | 1 | no |
| 3 | f | 48 | 29 | 5 | 66 | no | yes | 32 | 2 | 1 | 3 | 2 | 1 | 2 | 0 | 1 | yes |
| 4 | f | 44 | 42 | 2 | 5 | no | yes | 23 | 1 | 0 | 1 | 1 | 1 | 1 | 0 | 1 | no |
| 5 | f | 49 | 43 | 3 | 12 | yes | no | 23 | 2 | 1 | 1 | 1 | 1 | 1 | 0 | 0 | no |
| 6 | m | 57 | 49 | 3 | 24 | no | no | 19 | 1 | 2 | 1 | 0 | 1 | 1 | 0 | 0 | no |
| 7 | f | 53 | 40 | 3 | 36 | yes | no | 25 | 2 | 0 | 1 | 0 | 1 | 0 | 0 | 0 | no |
| 8 | f | 55 | 53 | 1 | 24 | no | yes | 23 | 1 | 1 | 1 | 1 | 1 | 0 | 0 | 0 | no |
| 9 | m | 67 | 64 | 1 | 30 | no | yes | 17 | 1 | 1 | 1 | 2 | 1 | 1 | 0 | 0 | no |
| 10 | f | 47 | 37 | 4 | 11 | no | yes | 28 | 3 | 1 | 1 | 2 | 1 | 1 | 0 | 0 | no |
| 11 | m | 55 | 45 | 4 | 3 | yes | yes | 27 | 1 | 1 | 1 | 0 | 1 | 0 | 0 | 0 | no |
| 12 | f | 61 | 54 | 4 | 9 | no | yes | 12 | 1 | 1 | 1 | 1 | 0 | 1 | 0 | 0 | no |
| 13 | m | 42 | 40 | 1 | 19 | yes | no | 25 | 1 | 0 | 1 | 0 | 1 | 1 | 0 | 0 | no |
| 14 | m | 56 | 55 | 1 | 11 | no | yes | 23 | 1 | 1 | 1 | 0 | 1 | 0 | 0 | 1 | no |
| 15 | f | 41 | 37 | 1 | 46 | no | no | 23 | 2 | 1 | 2 | 2 | 1 | 1 | 0 | 0 | no |
| 16 | f | 60 | 29 | 5 | 19 | no | no | 13 | 1 | 1 | 2 | 1 | 1 | 0 | 0 | 0 | no |
| 17 | m | 63 | 45 | 2 | 14 | no | yes | 18 | 0 | 1 | 1 | 0 | 1 | 1 | 0 | 0 | no |
| 18 | m | 55 | 45 | 3 | 10 | no | yes | 14 | 1 | 1 | 2 | 0 | 0 | 0 | 1 | 1 | no |
| 19 | f | 37 | 30 | 2 | 24 | yes | yes | 27 | 1 | 1 | 1 | 0 | 1 | 0 | 0 | 0 | no |
| 20 | f | 42 | 21 | 4 | 26 | no | yes | 22 | 2 | 1 | 1 | 1 | 1 | 2 | 0 | 0 | no |
| 21 | f | 34 | 21 | 2 | 12 | yes | yes | 29 | 0 | 0 | 1 | 0 | 0 | 0 | 0 | 1 | no |
| 22 | f | 45 | 39 | 3 | 12 | no | no | 21 | 1 | 1 | 1 | 1 | 1 | 0 | 0 | 0 | no |
| 23 | f | 46 | 35 | 3 | 19 | yes | yes | 21 | 1 | 1 | 1 | 0 | 1 | 0 | 0 | 1 | no |
| mean | 15f/8m | 50,7 | 40,8 | 2,8 | 31,0 | 6/17 | 15/8 | 21,9 | 1,3 | 0,9 | 1,4 | 0,8 | 0,9 | 0,7 | 0,2 | 0,4 | 1 yes/22 no |

**Supplemental Table 1A. Clinical characteristics and summary of pharmacological treatment history for each patient**

Pharmacological treatment history is based on Sackeim and colleagues[^1^](#_ENREF_1).

m = male, f = female in Gender column;

HDRS = Hamilton Depression Rating Scale 17 item; (5) Values in serotonin-reuptake inhibitors (SSRI), serotonin-noradernaline-reuptake inhibitors (SNRI), tricyclic antidepressants (TCA), Lithium, MAO-Inhibitor (Tranylcypromine) and Anti-epileptics columns represent number of different medications previously used in each category; (5) ECT = electroconvulsive therapy.
