# Supplementary material for: Bilateral ECT induces bilateral increases in regional cortical thickness
Source: Transl Psychiatry. 2016 Aug 23;6(8):e874–. doi: 10.1038/tp.2016.139 (PMC5022085; doi:10.1038/tp.2016.139)
Supplement: Supplementary Table 1B [file tp2016139x2.docx]

| Patient | Gender | Age  years | Electrode placement | Pulse width  msec | Number of sessions | HDRS | | Charge | | | Seizure duration | | |
| --- | --- | --- | --- | --- | --- | --- | --- | --- | --- | --- | --- | --- | --- |
|  |  |  |  |  |  | start | end | start  mC | end  mC | change  mC | start  sec | end  sec | change  sec |
| 1 | m | 55 | bl | 0,5 | 16 | 14 | 7 | 151,2 | 378,0 | 226,8 | 68 | 40 | -28 |
| 2 | F | 54 | bl | 0,5 | 12 | 24 | 3 | 302,4 | 504,0 | 201,6 | 43 | 15 | -28 |
| 3 | f | 48 | bl | 0,5 | 24 | 32 | 26 | 126,0 | 176,4 | 50,4 | 63 | 39 | -24 |
| 4 | f | 44 | bl | 0,5 | 7 | 23 | 5 | 100,8 | 176,4 | 75,6 | 51 | 29 | -22 |
| 5 | f | 49 | bl | 0,5 | 12 | 23 | 7 | 252,0 | 302,4 | 50,4 | 31 | 15 | -16 |
| 6 | m | 57 | bl | 0,5 | 14 | 19 | 6 | 403,2 | 1008,0 | 604,8 | 68 | 39 | -29 |
| 7 | f | 53 | bl | 0,5 | 13 | 25 | 15 | 151,2 | 554,4 | 403,2 | 54 | 26 | -28 |
| 8 | f | 55 | bl | 0,5 | 21 | 23 | 8 | 151,2 | 604,8 | 453,6 | 48 | 27 | -21 |
| 9 | m | 67 | bl | 0,5 | 14 | 17 | 6 | 378,0 | 756,0 | 378,0 | 32 | 33 | 1 |
| 10 | f | 47 | bl | 0,5 | 16 | 28 | 9 | 151,2 | 151,2 | 0 | 61 | 46 | -15 |
| 11 | m | 55 | bl | 0,5 | 19 | 27 | 26 | 302,4 | 504,0 | 201,6 | 62 | 43 | -19 |
| 12 | f | 61 | bl | 0,5 | 14 | 12 | 15 | 151,2 | 151,2 | 0 | 36 | 46 | 10 |
| 13 | m | 42 | bl | 0,5 | 16 | 25 | 20 | 100,8 | 302,4 | 201,6 | 49 | 39 | -10 |
| 14 | m | 56 | bl | 0,5 | 30 | 23 | 9 | 151,2 | 302,4 | 151,2 | 47 | 44 | -3 |
| 15 | f | 41 | bl | 0,5 | 7 | 23 | 16 | 151,2 | 453,6 | 302,4 | 24 | 35 | 11 |
| 16 | f | 60 | bl | 0,5 | 12 | 13 | 20 | 126,0 | 126,0 | 0 | 34 | 30 | -4 |
| 17 | m | 63 | bl | 0,5 | 32 | 18 | 7 | 453,6 | 806,4 | 352,8 | 48 | 30 | -18 |
| 18 | m | 55 | bl | 0,5 | 24 | 14 | 14 | 75,6 | 604,8 | 529,2 | 71 | 55 | -16 |
| 19 | f | 37 | bl | 0,5 | 19 | 27 | 10 | 50,4 | 453,6 | 403,2 | 67 | 30 | -37 |
| 20 | f | 42 | bl | 0,5 | 18 | 22 | 21 | 50,4 | 277,2 | 226,8 | 78 | 46 | -32 |
| 21 | f | 34 | bl | 0,5 | 18 | 29 | 13 | 25,2 | 100,8 | 75,6 | 67 | 39 | -28 |
| 22 | f | 45 | bl | 0,5 | 13 | 21 | 5 | 201,6 | 277,2 | 75,6 | 45 | 34 | -11 |
| 23 | f | 46 | bl | 0,5 | 18 | 21 | 22 | 25,2 | 75,6 | 50,4 | 59 | 50 | -9 |
| mean | 15f/8m | 50,7 | 23 bl | 0,5 | 16,9 | 21,9 | 12,6 | 175,3 | 393,4 | 218,0 | 52,4 | 36,1 | -16,3 |

**Supplemental Table 1B. Details of ECT treatment for each patient**

f female, m male, bl bilateral mC microCoulomb,

Seizure duration: duration of seizure based on cuff method
