# Supplementary material for: Bilateral ECT induces bilateral increases in regional cortical thickness
Source: Transl Psychiatry. 2016 Aug 23;6(8):e874–. doi: 10.1038/tp.2016.139 (PMC5022085; doi:10.1038/tp.2016.139)
Supplement: Supplementary Table 2 [file tp2016139x3.docx]

|  | Mean increase in thickness after ECT in mm (n=19) | Mean increase in thickness after ECT in reponders in mm (n=10) | Mean increase in thickness after ECT in non-responders in mm (n=9) | Difference between responder and non-responders  p^1^ |
| --- | --- | --- | --- | --- |
| *Left hemisphere* |  |  |  |  |
| Superior temporal cortex | 0,09 | 0,10 | 0,08 | 0,61 |
| Middel temporal cortex | 0,10 | 0,10 | 0,09 | 0,95 |
| Inferior temporal cortex | 0,11 | 0,13 | 0,09 | 0,34 |
| Temporal pole | 0,25 | 0,28 | 0,21 | 0,32 |
| Insula | 0,10 | 0,14 | 0,06 | **0,017** |
| *Right hemisphere* |  |  |  |  |
| Superior temporal cortex | 0,09 | 0,10 | 0,09 | 0,98 |
| Middel temporal cortex | 0,09 | 0,09 | 0,09 | 0,95 |
| Inferior temporal cortex | 0,08 | 0,10 | 0,05 | 0,26 |
| Temporal pole | 0,27 | 0,29 | 0,25 | 0,73 |
| Insula | 0,09 | 0,13 | 0,06 | **0,017** |

**Supplemental Table 2**

Mean increases in cortical thickness of predefined cortical parcellations (from the Desikan-Killiany Atlas) after ECT, for the whole group (n=19) and separate for responders (n=10) and non-responders (n=9)

^1^ independent t-test
